# Detection of methylated *BCAT1* and *IKZF1* after curative‐intent treatment as a prognostic indicator for colorectal cancer recurrence

**DOI:** 10.1002/cam4.5008

**Published:** 2022-07-13

**Authors:** Susanne K. Pedersen, Erin L. Symonds, Amitesh C. Roy, Kathryn J. Cornthwaite, Lawrence C. LaPointe, Graeme P. Young

**Affiliations:** ^1^ Flinders Health and Medical Research Institute Flinders University South Australia Australia; ^2^ Clinical Genomics Inc New Jersey USA; ^3^ Bowel Health Service Flinders Medical Centre South Australia Australia; ^4^ Department of Medical Oncology Flinders Medical Centre South Australia Australia

**Keywords:** *BCAT1*, circulating tumor DNA, colorectal cancer, *IKZF1*, methylation, recurrence

## Abstract

**Background:**

The risk of recurrence after completion of curative‐intent treatment of colorectal cancer (CRC) is hard to predict. Post‐treatment assaying for circulating tumor DNA (ctDNA) is an encouraging approach for stratifying patients for therapy, but the prognostic value of this approach is less explored. This study aimed to determine if detection of methylated *BCAT1* and *IKZF1* following completion of initial treatment identified patients with a poorer recurrence‐free survival (RFS).

**Methods:**

142 CRC stage I‐III cases with at least 2 years of follow up (unless recurrence was evident sooner) and a methylated *BCAT1/IKZF1* test result between 2 weeks and 12 months after completion of initial treatment were eligible for study inclusion. The association between *BCAT1/IKZF1* and RFS was assessed by the log‐rank (Mantel‐Cox) method. Cox proportional hazard regression analysis was used for multivariable survival analysis.

**Results:**

Thirty‐three (23.2%) had recurrence at a median 1.6y (interquartile range: 0.8–2.4). Methylated *BCAT1/IKZF1* was detected in 19 of the 142 patients (13.4%) and was associated with a significant risk of recurrence (hazard ratio [HR] 5.7, 95%CI: 1.9–17.3, *p* = 0.002). Three‐year RFS for patients with or without detectable methylated *BCAT1/IKZF1* was 56.5% and 83.3%, respectively. Multivariable analysis showed that detection of methylated *BCAT1/IKZF1* (HR = 2.6, *p* = 0.049) and site of the primary tumor (HR = 4.2, *p* = 0.002) were the only significant prognostic indicators of poor RFS.

**Conclusions:**

*BCAT1/IKZF1* methylation testing after curative‐intent treatment is an independent prognostic indicator for RFS and identifies a subgroup at high risk. Personalized surveillance is warranted for patients with these ctDNA biomarkers detectable after curative‐intent treatment.

## Graphical abstract

Detection of circulating DNA methylated in *BCAT1/IKZF1* after curative‐intent treatment is an independent prognostic indicator for recurrence.

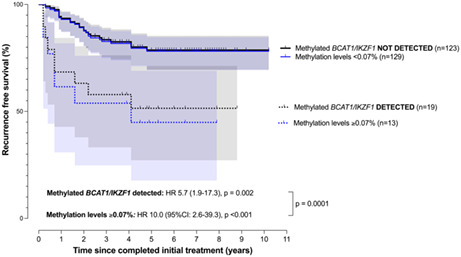

## 1 INTRODUCTION

Despite advances in therapy, the cure rates and long‐term survival from colorectal cancer (CRC) have not changed significantly in recent decades; CRC remains a leading cause of cancer‐associated deaths worldwide.^1^ CRC prognosis is linked to the tumor‐node‐metastasis (TNM) classification and effectiveness of initial treatment. Surgical resection with additional chemotherapy and/or radiation therapy, given either in the neoadjuvant or adjuvant setting to eradicate minimal disease, remains the standard of care.^2^, ^3^, ^4^ Unfortunately, about 25–40% of patients treated for CRC with curative intent still suffer recurrence within five years of initial treatment, with most occurring within the first three.^5^, ^6^

The characteristics of patients who remain at risk of recurrence after initial curative‐intent treatment are hard to predict. Treatment inadequacy only becomes evident when recurrence is detected, primarily by radiographic imaging, during stage‐dependent surveillance protocols.^7^, ^8^, ^9^, ^10^, ^11^ Excessive surveillance may result in unnecessary radiation exposure, increased medical costs and unnecessary patient anxiety, while infrequent follow‐up may result in missing recurrences whilst still curable.^12^, ^13^

The high inter‐patient and intra‐tumor heterogeneity in CRC makes implementation of a personalized surveillance regimen based on a patient's risk of recurrence challenging.^14^ There is a need for biomarkers that predict prognosis of CRC patients after completing initial curative‐intent treatment to improve overall CRC survival. This would aid stratification of those who may benefit from prolonged/enhanced surveillance and/or additional therapy despite being apparently cancer‐free after initial treatment. Detection of circulating tumor DNA (ctDNA) by assaying cell‐free DNA (cfDNA) for somatic mutations is now well‐established in cancer research. Studies suggest that there is a high risk of CRC recurrence if somatic ctDNA biomarkers are detected following surgical resection and/or prior to adjuvant therapy.^15^, ^16^, ^17^, ^18^, ^19^

Targeting CRC specific epigenetic changes improves the ctDNA detection rate as these methylation changes occur more commonly than the most frequently targeted mutations.^20^, ^21^, ^22^, ^23^, ^24^, ^25^, ^26^ For example, 99% of CRC tissues are hypermethylated in *BCAT1* and *IKZF1*.^27^ In contrast, the most common mutations in CRC are only found in approximately 50% of the tumor tissues.^28^ Hence, tumor genotyping is not required if these two methylation biomarkers are used for ctDNA detection, and testing for methylated *BCAT1/IKZF1* ctDNA is unlikely to be confounded by tumor heterogeneity or clonal drift following adjuvant therapy. Detection of circulating methylated *BCAT1/IKZF1* DNA is associated with primary CRC; the biomarkers disappear following adequate CRC treatment; their detection during surveillance is indicative of CRC recurrence for which we have previously reported a 63–68% sensitivity and 92–98% specificity; and the ctDNA test is more sensitive than CEA testing.^27^, ^29^, ^30^, ^31^, ^32^, ^33^, ^34^

We report a prospective longitudinal observational study aimed to determine if *BCAT1/IKZF1* methylation testing of patients, who had completed initial treatment with curative intent for CRC stage I‐III, could identify patients most likely to develop recurrence.

## 2 METHODS

### 2.1 Study overview

The study cohort was drawn from an observational clinical trial enrolling cases (≥18 years) being treated for primary adenocarcinoma of the colon or rectum and undergoing prospective surveillance for recurrence. Cases were eligible for this study if they had completed initial treatment with curative intent for primary CRC stages I‐III; had at least two years of monitoring (unless recurrence was diagnosed sooner, or death intervened) with at least one radiographic examination; and had a methylation *BCAT1/IKZF1* blood result in the year following completion of initial treatment and where blood was collected at, or adjacent to, a standard surveillance visit. The primary outcome measure was recurrence‐free survival. The study aimed to determine whether detection of methylated *BCAT1/IKZF1* within 0.5–12 months after completing initial treatment identified patients at elevated risk of CRC recurrence. The clinical trial was approved by the Southern Adelaide Clinical Human Research Ethics Committee (ethics number 134.045), registered at the Australian New Zealand Clinical Trials Registry (12611000318987) and conducted in accordance with the Declaration of Helsinki (WMA 2013) and Good Clinical Practice (GCP ICH‐E6). Written informed consent was obtained from all subjects prior to any procedures.

### 2.2 Study population

Patients diagnosed with invasive stage I‐III CRC were eligible for study consideration. Treatment plans were implemented according to site and stage of the primary tumor, according to relevant professional guidelines and clinical standard of care. Surveillance monitoring comprised regular clinical assessments and radiographic imaging according to applicable guidelines at the Southern Adelaide Local Health Network in the period March 2005 to December 2021. Inclusion criteria comprised at least two years of physician‐directed monitoring after primary curative‐intent treatment and provision of a blood sample for *BCAT1/IKZF1* methylation testing no earlier than 2 weeks, and within 12 months, of completing initial treatment. Patient demographics, histopathology, treatment and imaging details were documented. TNM and AJCC staging (AJCC guidelines version 8) were confirmed through clinicopathological findings at surgery for colon and upper to mid‐rectal cancers.^35^ For those with low rectal tumors, staging was based on pre‐treatment magnetic resonance imaging (MRI), if neoadjuvant therapy was given. If synchronous cancers were documented, the staging of the most advanced lesion was used as the primary diagnosis. Radiographic examination of chest, abdomen, and pelvis were performed at 12‐month intervals subject to the discretion of the clinician. The presence or absence of clinically apparent recurrence was determined based on findings of diagnostics tests (computed tomography (CT), positron emission tomography (PET) or MRI imaging; or colonoscopy) as previously detailed.^29^ Recurrence was defined as distant for lesions in another in another organ, non‐regional lymph nodes, or the peritoneal cavity, while lesions present at the site of anastomosis or in draining lymph nodes where defined as locoregional recurrence. Recurrence was classified as distant if both local and distant recurrences were documented. Exclusion criteria included cases with stage 0 or stage IV CRC, inadequate staging, failure to meet blood sampling requirements, incomplete treatment, a surveillance period of less than 2 years (except where recurrence was diagnosed or death from CRC intervened), and/or inadequate radiographic imaging within the first 2 years of surveillance. Cases were also excluded if diagnosed with other cancers or metachronous (new primary) CRC during surveillance.

### 2.3 *BCAT1* / *IKZF1* methylation testing of circulating cell‐free DNA

cfDNA was extracted from plasma, bisulphite converted and analyzed in a real‐time polymerase chain reaction (PCR) assay simultaneously detecting methylation in targeted regions in *BCAT1* and *IKZF1*, and a CpG‐free target region in *ACTB* as described previously.^36^ If at least one of the three PCR replicates had an amplification signal, such sample was annotated as ‘methylated *BCAT1/IKZF1* detected’. The mass of DNA methylated in *BCAT1/IKZF1* was expressed as percent of cfDNA, and samples with levels of 0.07% or more was considered outside of the normal upper reference limit (URL) and hence deemed positive.^37^

### 2.4 Statistical methods

Descriptive statistics were used for baseline characteristics. Mann–Whitney (rank sum) and Fisher Exact testing were applied for categorical variables and continuous variables, respectively. The primary outcome measure was recurrence‐free survival, measured from the date of completing initial treatment with curative intent to documented first clinically apparent recurrence and was censored at the last known follow‐up time point. The log‐rank test (Mantel‐Cox) method was used for association of detection of circulating DNA methylated in *BCAT1/IKZF1* with recurrence free survival. A Cox power analysis was performed using *n* = 142, hazard ratio (HR) of 5.7, standard deviation (SD) = 0.343 and a probability of recurrence = 0.237, estimating a power of 93.4% (Stata version 17.0). Multivariable survival analysis by Cox Proportional Hazards (Cox PH) modeling was performed using the following covariates: detection of methylated *BCAT1/IKZF1* DNA post‐treatment, age, gender, T‐stage, N‐stage, extra‐ and/or intra‐mural invasion (EMVI/IMVI), lymphovascular invasion, perineural invasion, differentiation, location of primary CRC and nature of treatment. A Cox power analysis, using HR 2.8, STDEV = 0.507 and a probability of recurrence = 0.222 for the subset available for multivariable analysis (*n* = 108), estimated a power of 72.4% (Stata version 17.0). The Clopper Pearson and Babtista‐Pike methods were used for calculations of 95% confidence intervals (CI) and odds ratios (OR), respectively. All statistical tests were 2‐sided. *p*‐values <0.05 were considered significant. GraphPad Prism version 9.3.1 was used for statistical analyses unless stated otherwise.

## 3 RESULTS

### 3.1 Study population

**FIGURE 1:**
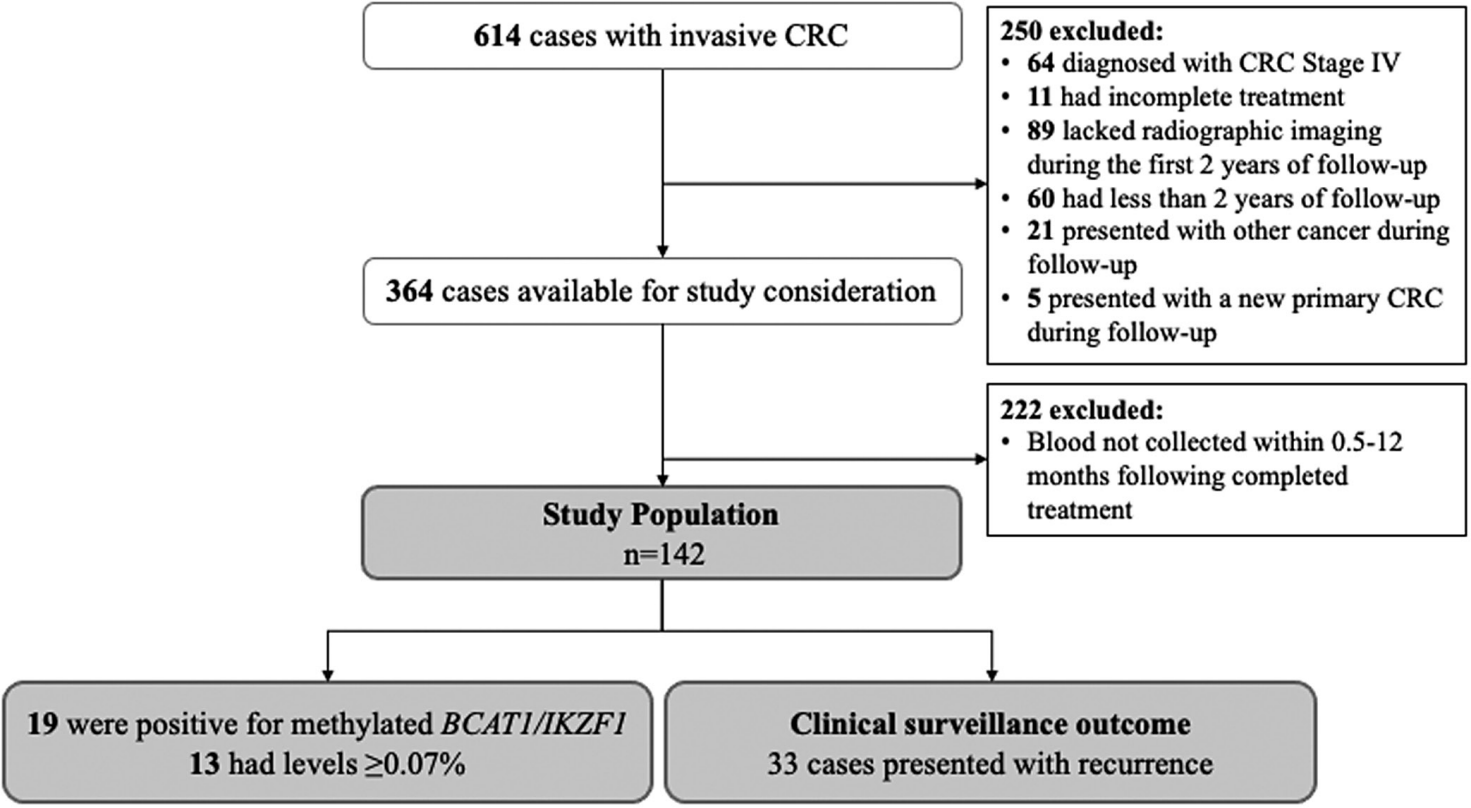
Disposition of study cases.

Of the 614 CRC patients enrolled, 250 were excluded for failure to meet study inclusion criteria, and an additional 222 patients were excluded for lack of blood collection within 0.5–12 months after completion of initial treatment, leaving 142 eligible patients (Figure 1).

Demographic, clinical features, nature of treatment and outcome during surveillance of the eligible 142 cases are detailed in Table 1. At the time of CRC diagnosis, the median age of the study population was 63.9 years (min‐max: 30–83 years), 60.6% were males, and 23.2%, 38.0% and 38.7% had stage I, II and III disease, respectively. The majority (70.4%) had primary colon cancer while the rest had primary rectal cancer.

**TABLE 1:** Demographic, clinical features and outcome during surveillance

Characteristics	Cohort	CRC recurrence		*p* ^a^
		Yes	No	
**Cases, *N* (%)**	**142**	**33**	**109**	**–**
Median age at diagnosis, years (IQR)	63.9	62.9 (56.2–82.0)	64.8 (56.2–69.8)	0.934
Males, *n* (%)	86 (60.6)	25 (75.8)	61 (56.0)	**0.041**
Location, *n* (%)				
Colon	100 (70.4)	17 (51.5)	83 (76.1)	**<0.01**
Rectum	42 (29.6)	16 (48.5)	26 (23.9)	
Histopathology, *n*/total (%)				
EMVI and/or IMVI	19/113 (16.8)	9/27 (33.3)	10/86 (11.6)	**<0.01**
Lymphovascular invasion	39/129 (30.2)	16/29 (55.2)	23/100 (23.0)	**<0.01**
Perineural invasion	20/129 (15.5)	9/28 (32.1)	11/101 (10.9)	**<0.01**
AJCC stage, *n* (%)				
I	33 (23.2)	2 (6.1)	31 (28.4)	**<0.01**
II	54 (38.0)	10 (30.3)	44 (40.4)	0.298
III	55 (38.7)	21 (63.6)	34 (31.2)	**<0.01**
Nature of initial treatment, *n* (%)				
Surgery only	74 (52.1)	8 (24.2)	66 (60.6)	**<0.01**
Neo‐adjuvant therapy only	6 (4.2)	4 (12.1)	2 (1.8)	**0.010**
Neo‐adjuvant therapy and surgery	6 (4.2)	3 (9.1)	3 (2.8)	0.112
Surgery and adjuvant chemotherapy	41 (28.9)	13 (39.4)	28 (25.7)	0.129
Neo‐adjuvant therapy, surgery and adjuvant chemotherapy	15 (10.6)	5 (15.2)	10 (9.2)	0.327
**Median follow‐up, years (IQR)**	4.2 (2.7–6.5)	1.6 (0.8–2.4)	5.3 (3.7–6.9)	**<0.01**
**CRC‐related death, *n* (%)**	13 (9.2)	13 (39.4)	–	–

Abbreviations: AJCC: American Joint Committee on Cancer; CRC: colorectal cancer; IQR: interquartile range; EMVI, extramural vascular invasion; IMVI, intramural vascular invasion.

^a^ Mann‐Whitney and Fisher Exact testing were applied for categorial variables and continuous variables, respectively.

### 3.2 Clinical outcome

Median follow‐up time after completing initial treatment was 4.2 years (interquartile range, IQR: 2.7–6.5). The surveillance outcome (i.e., recurrence status) by patient characteristics and nature of initial treatment is summarized in Table 1.

Thirty‐three (23.2%) of the patients had a recurrence at a median 1.6 years (IQR: 0.8–2.4) after completion of curative‐intent treatment. Twenty‐one (63.6%), ten (30.3%) and two (6.1%) of the recurrences occurred in patients treated for CRC stage III, II and I, respectively. Twenty‐two (66.7%) recurrences occurred at distant sites – liver (*n* = 7, 31.8%), lung (*n* = 6, 27.3%), distant nodes (*n* = 5, 22.7%) and peritoneum (*n* = 4, 18.2%) – while the remaining 11 (33.3%) were local. Median follow‐up time for those who remained recurrence‐free after completion of curative‐intent treatment was 5.3 years (*n* = 109, IQR: 3.7–6.9).

### 3.3 Baseline characteristics according to post‐treatment detection of methylated *BCAT1* / *IKZF1*

Circulating DNA methylated in *BCAT1/IKZF1* was detected in 19 (13.2%) patients following completion of initial treatment with curative‐intent. Detection of methylated *BCAT1/IKZF1* was significantly associated with recurrence, CRC‐related death, nature of initial treatment and perineural invasion, but not with stage, location of primary tumor, age, gender or extra−/intra−/lympho‐ vascular invasion, Table 2. The time elapsed between treatment completion and *BCAT1/IKZF1* methylation testing was not significantly different between those who had recurrence and those who remained recurrence‐free (median 2.4 months (IQR: 1.7–5.0) versus 3.1 months (IQR: 1.8–4.9), *p* = 0.529).

**TABLE 2:** Relationship between detection of methylated *BCAT1/IKZF1* DNA post curative‐intent treatment and patient characteristics

Characteristics	Total	Methylated *BCAT1/IKZF1*		
		Not detected	Detected	*p* ^a^
**Cases, N**	**142**	**123**	**19**	**–**
Age at diagnosis, median years (IQR)	–	65.2 (53.6–73.1)	62.7 (58.4–64.8)	0.557
Males, *n* (%)	86 (60.6)	73 (59.4)	13 (68.4)	0.453
AJCC Stage, *n* (%)				
I	33 (23.2)	31 (25.2)	2 (10.5)	0.159
II	54 (38.0)	48 (39.0)	6 (31.6)	0.535
III	55 (38.7)	44 (35.7)	11 (57.9)	0.066
Location, *n* (%)				
Colon	100 (70.4)	90 (73.2)	10 (52.6)	0.067
Rectum	42 (29.6)	33 (26.8)	9 (47.4)	
Histopathology, *n*/total (%)				
EMVI and/or IMVI	19/113 (16.8)	14/96 (14.5)	5/17 (29.4)	0.131
Lymphovascular invasion	39/129 (30.2)	31/113 (27.4)	8/16 (50.0)	0.066
Perineural invasion	20/129 (15.5)	14/112 (12.5)	6/17 (35.3)	**0.016**
Nature of initial treatment, *n* (%)				
Surgery only	74 (52.1)	69 (56.1)	5 (26.3)	**0.016**
Neo‐adjuvant therapy only	6 (4.2)	6 (4.9)	0 (0)	0.327
Neo‐adjuvant therapy and surgery	6 (4.2)	5 (4.1)	1 (5.3)	0.810
Surgery and adjuvant chemotherapy	41 (28.9)	32 (26.0)	9 (47.4)	0.056
Neo‐adjuvant therapy, surgery and adjuvant chemotherapy	15 (10.6)	11 (8.9)	4 (21.1)	0.110
**Time between completion of treatment and** ‐**methylation testing, median months (IQR)**		3.1 (1.8–5.3)	2.0 (1.4–4.3)	0.154
Clinical outcome during surveillance, *n* (%)				
Recurrence	33 (23.2)	24 (19.5)	9 (47.4)	**0.007**
CRC‐related death	13 (9.2)	8 (5.6)	5 (26.3)	**0.005**

Abbreviations: AJCC: American Joint Committee on Cancer; CRC: colorectal cancer; IQR: interquartile range; EMVI, extramural vascular invasion; IMVI, intramural vascular invasion.

^a^ Mann‐Whitney and Fisher Exact testing were applied for categorical variables and continuous variables, respectively.

### 3.4 Detection of methylated *BCAT1* / *IKZF1* and subsequent risk of recurrence

**FIGURE 2:**
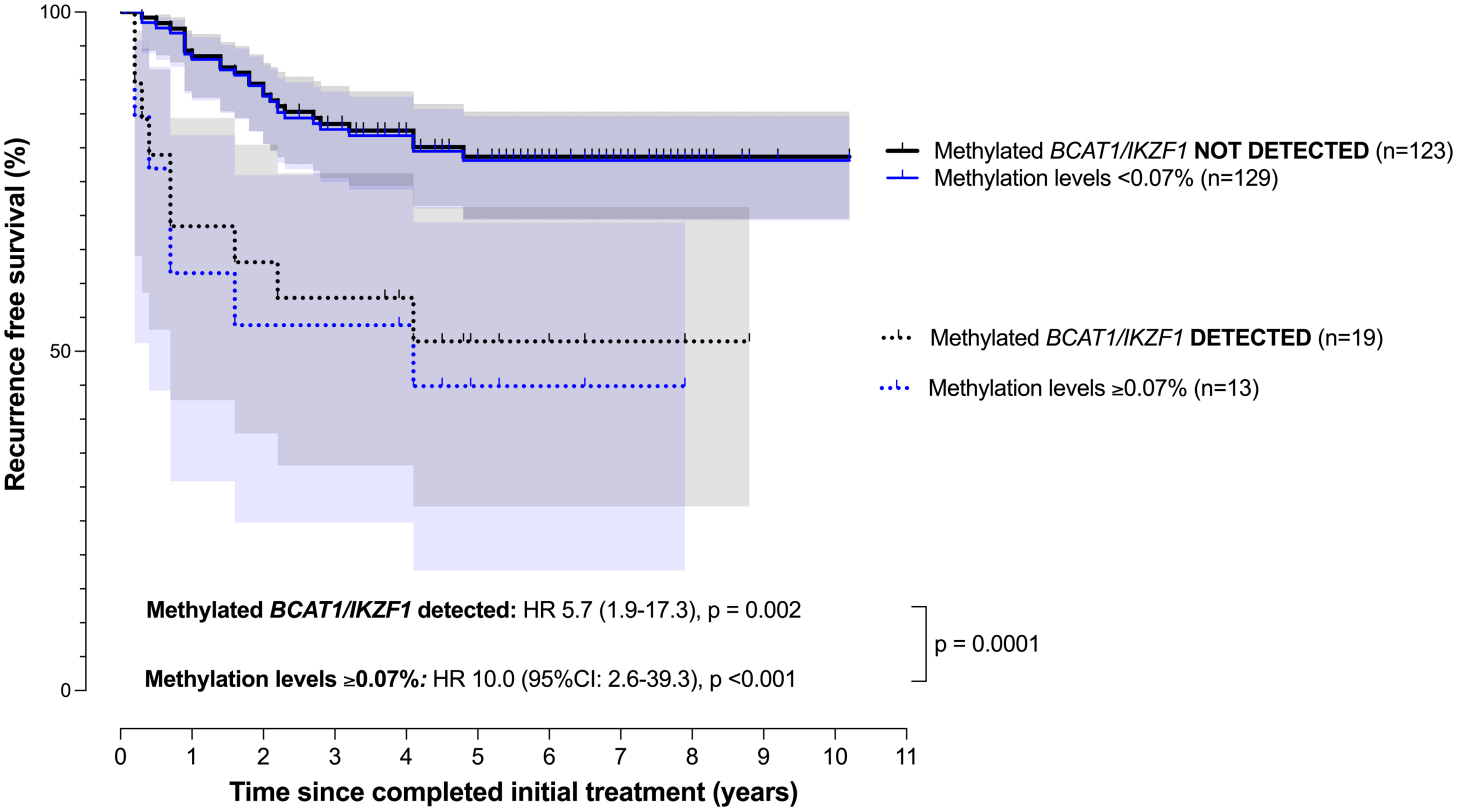
Recurrence free survival analysis stratified by detection of methylated *BCAT1/IKZF1* following completion of initial treatment (time 0). HR: hazard ratio. Survival‐curves are based on detection of methylated *BCAT1* and/or *IKZF1* (black lines; 95% CI band in gray shading) and for those with methylation levels of 0.07% or more (blue lines; 95% CI band in light blue shading).

Risk of recurrence was higher when methylation in *BCAT1* and/or *IKZF1* was detected (*n* = 19, HR 5.7 [95% CI: 1.9–17.3], *p* = 0.002). Considering risk according to levels of methylation of 0.07% or more (*n* = 13), the HR was 10.0 (95%CI: 2.6–39.3, *p* < 0.001), Figure 2. This HR estimate was significantly higher compared to the HR estimate associated with any detectable signal (*p* = 0.0001). The 3‐year recurrence‐free survival for patients with or without detectable circulating DNA methylated in *BCAT1/IKZF1* after completion of treatment was 56.5% and 83.3%, respectively, and 49.3% for those who had methylation levels of 0.07% or more.

### 3.5 Predictors of recurrence

Cox proportional hazards regression was used to estimate the effect of variables with respect to predicting recurrence‐free survival, Table 3. Detection of circulating DNA methylated in *BCAT1/IKZF1* post‐treatment with curative intent (HR = 2.6, *p* = 0.049) and a rectal location of the primary tumor (HR = 4.2, *p* = 0.002) remained significant prognostic factors for poor recurrence‐free survival after adjusting for all potential confounders.

**TABLE 3:** Predictors of risk of recurrence

Covariates	*N*	Univariate		Multivariable^a^	
		HR (95% CI)	*p*	HR (95% CI)	*p*
Age^b^	142	1.0 (1.0–1.1)	0.840	–	–
Female vs. male	142	2.2 (1.0–5.2)	0.053	–	–
Location (colon vs. rectum)	142	2.5 (1.3–5.0)	**0.008**	4.2 (1.7–10.6)	**0.002**
Differentiation (well or moderate vs. poor)	124	1.8 (0.7–4.2)	0.174	–	–
T‐stage (T1‐2 vs. T3‐4)	142	3.8 (1.5–12.8)	**0.013**	2.8 (0.8–13.5)	0.152
N‐stage (N0 vs. N1‐2)	142	3.5 (1.8–7.0)	**<0.001**	1.4 (0.5–4.0)	0.547
Intra‐ and/or extra‐mural vascular invasion (no/yes)	113	3.3 (1.4–7.1)	**0.004**	0.7 (0.2–2.3)	0.589
Lymphovascular invasion (no/yes)	129	3.7 (1.8–7.8)	**<0.001**	1.6 (0.6–4.6)	0.326
Perineural invasion (no/yes)	129	3.5 (1.5–7.4)	**0.002**	2.6 (0.8–7.4)	0.087
Treatment included surgery (no/yes)	142	2.5 (1.3–5.3)	**0.010**	0.7 (0.1–4.2)	0.786
Methylated *BCAT1/IKZF1* detected (no/yes)	142	3.1 (1.4–6.5)	**0.004**	2.6 (0.9–6.5)	**0.049**

Abbreviation: HR, hazard ratio.

^a^ The multivariable cox proportional hazard regression modeling only included co‐variates identified to be significant in the univariate analysis and cases with missing data were omitted from multivariable analysis (*n* = 13). In the resulting sub‐population (*n* = 108), 24 had recurrence.

^b^ Analyzed as continuous covariate.

In patients who had recurrence, detection of methylated *BCAT1/IKZF1* was significantly associated with the time elapsed since completion of curative‐intent treatment and methylation testing, but not with age, gender, T‐stage, N‐stage, intra/extramural invasion, nature of treatment, and the time elapsed between methylation testing and recurrence, Table 4. Similar significance levels for each characteristic were also observed on use of a 0.07% methylation threshold for assay positivity (data not shown).

**TABLE 4:** Characteristics of cases who had recurrence according to detection or absence of methylated *BCAT1/IKZF1*

Clinical characteristic	Methylated *BCAT1/IKZF1* detected		*p*
	No (*n* = 24)	Yes (*n* = 9)	
Age, median years (IQR)	63.6 (54.8–70.6)	62.6 (60.0–70.9)	0.914
Males, *N* (%)	18 (75.0)	7 (77.8)	1.000
T3‐4 stage, *N* (%)	22 (91.7)	7 (77.8)	0.276
N1‐2 stage, *N* (%)	6 (25.0)	3 (33.3)	0.631
Histopathology, *n*/total (%)			
EMVI and/or IMVI	5/19 (26.3)	4/8 (50.0)	0.234
Lymphovascular invasion	10/22 (45.5)	6/7 (85.7)	0.061
Perineural invasion	5/21 (23.8)	4/7 (57.1)	0.101
Initial treatment with curative intent, *N* (%)			
Surgery only	6 (25.0)	2 (22.2)	1.000
Neoadjuvant only	4 (16.7)	0	0.312
Neoadjuvant and surgery	2 (8.3)	1 (11.1)	1.000
Surgery and adjuvant chemotherapy	9 (37.5)	4 (44.4)	1.000
Neoadjuvant, surgery and adjuvant chemotherapy	3 (12.5)	2 (22.2)	0.597
Median months (IQR) elapsed between			
Completed treatment and methylation testing	3.1 (1.9–5.4)	1.7 (1.2–2.3)	**0.007**
Methylation testing and CRC recurrence	19.7 (8.8–24.4)	4.7 (2.0–21.6)	0.065

Abbreviations: EMVI, extramural vascular invasion; IMVI, intramural vascular invasion; IQR, interquartile range.

## 4 DISCUSSION

This observational study showed that detection of circulating DNA methylated in *BCAT1/IKZF1* following completion of curative‐intent treatment for CRC stage I‐III was associated with poorer recurrence‐free survival. The estimated 3‐year recurrence‐free survival for patients with or without detectable circulating DNA methylated in *BCAT1/IKZF1* post‐treatment was 56.5% and 83.3%, respectively. The estimated hazards ratios for recurrence were high; risk rose from 5.7 times more likely to experience recurrence when any signal was detected to 10.0 if a positivity threshold of 0.07% methylation was used. Detection of methylated *BCAT1/IKZF1* after completion of curative‐intent treatment was predictive of recurrence, independent of established pre‐treatment predictors of stage and certain histopathological features.

Nature of initial treatment is driven primarily by stage and certain other clinicopathological features, including location, all of which are only modest predictors of recurrence risk.^38^, ^39^, ^40^, ^41^, ^42^ Biomarkers assayed after completing initial curative‐intent treatment would be expected to be more useful than pre‐treatment variables as they are assessing risk after initial curative‐intent treatment has been completed, and as such would be expected to represent residual factors of importance in contrast to reflecting the clinical state prior to modification by treatment. Our findings that methylated *BCAT1/IKZF1* DNA identified a subgroup with a risk for recurrence ranging between 5 and 10, was higher than recurrence‐free survival risk which has been reported using somatic‐mutation based ctDNA markers.^43^, ^44^, ^45^ These studies have generally reported a 2‐ to 3‐fold increase in risk of recurrence. There are other studies reporting on the prognostic value of methylation‐based detection of ctDNA but their estimates arose from testing before commencing treatment.^46^, ^47^

At this point in time, there is no gold‐standard for diagnosing the presence of minimal residual disease (MRD) and we are dependent on subsequent clinical course to identify those cases where disease has not been eradicated. There is a considerable amount of literature reporting the utility of somatic mutation‐ and/or epigenetic‐based detection of ctDNA after surgical resection to identify those patients might benefit the most from adjuvant chemotherapy.^15^, ^18^, ^25^, ^48^, ^49^ While the application of somatic‐mutation marker testing after surgery (but prior to schedule adjuvant therapy) has been shown to be useful, once chemo‐therapy has been given, clonal shifts might occur.^50^ Methylation ctDNA markers, such as those used here, are characteristic of a large proportion of CRC and quite possibly not subject to clonal shifts as a result. It would therefore seem most logical to apply epigenetic based detection of ctDNA to cases after curative‐intent therapy has been completed (as was done in the current study) as some pre‐treatment predictors such as stage are modified by treatment and could become less predictive relative to those applied after completion. This is supported by the current study which demonstrated that post‐treatment detection of methylated *BCAT1/IKZF1* DNA was prognostic independent of stage at diagnosis and other established pre‐treatment prognostic predictors. As detection of methylated *BCAT1/IKZF1* after completion of curative‐intent treatment was an independent prognostic predictor, this points to the likely value of adapting case management in those who are positive after treatment.

Our prior publications have focused on using the methylated *BCAT1/IKZF1* ctDNA test for indication of radiographic‐apparent recurrence and compare it to CEA.^27^, ^34^, ^37^ As such it performed better than CEA, but those studies did not examine long‐term follow‐up to determine if an apparent false‐positive ctDNA test result did indeed indicate presence of disease not yet apparent by imaging. This study was therefore undertaken to explore the prognostic value of methylated *BCAT1/IKZF1* ctDNA testing after completion of initial treatment with curative intent, since if the ctDNA test predicts disease‐free survival, then the most obvious explanation for this ctDNA marker panel being prognostic is that it is a marker of MRD. Here, we demonstrated that detection of circulating methylated *BCAT1/IKZF1* DNA is indicative of the presence of tumor even if it is not radiographic apparent.

In the 33 patients who had recurrence during surveillance, the time elapsed between completion of curative‐intent treatment and *BCAT1/IKZF1* methylation testing was statistically different in the nine who were *BCAT1/IKZF1* positive (median 1.7 months) compared to the 24 who were not (median 3.1 months, *p* = 0.007). The timing of somatic‐mutation based ctDNA testing after surgery has previously been reported, but that pertained to ctDNA testing performed within 4–10 weeks of surgical resection.^15^, ^16^, ^18^ Our study provides some insight into timing of methylated *BCAT1/IKZF1* testing relative to completion of curative‐intent treatment, but more detailed exploration is warranted. The time elapsed between *BCAT1/IKZF1* methylation testing and recurrence was not significantly different in recurrence cases who were *BCAT1/IKZF1* positive compared to those who were not. Larger prospective trials with defined blood sampling intervals are required to clarify the best time for methylated *BCAT1/IKZF1* DNA testing – ideally with comparison to somatic ctDNA markers.

Based on these results, several clinical responses to post‐treatment detection of methylated *BCAT1/IKZF1* must be considered. The first is personalization of surveillance to increase its intensity, a strategy that would be justified in stage I and stage IIA cases. Personalization through continuance of intensive surveillance beyond three years would also seem justified. Note that some positive cases experienced recurrence more than three years after completion of treatment (Figure 2). Our results show that incorporation of ctDNA testing based on detection of methylated *BCAT1/IKZF1* after surgical resection and/or after completion of curative‐intent treatment into similar studies is now warranted.

This study has some limitations as cases was drawn from an observational clinical trial conducted in a usual‐care clinical setting where follow‐up protocols were subject to a modest degree of variance according to physician practice and the timing of post‐treatment blood sampling was not standardized. However, variations in such timing were not seen to influence the results in the multivariable analysis. While the studied cases were heterogeneous, being of colonic and rectal origin, of three stages, and of varying treatment strategies, it was considered important to demonstrate applicability across the clinical contexts applicable to CRC. It should be noted that two stage I cases had recurrence and both were predicted by detection of methylated *BCAT1/IKZF1*. It was not possible to undertake separate survival analyses in cases with colon or rectal cancer as there were insufficient events to do this. Larger studies are needed to be certain that the magnitude of risk applies equally to both cancers.

Risk for recurrence was shown to be dependent on the level of methylated *BCAT1/IKZF1* in plasma. A recent study has shown that application of an URL for test positivity improves specificity when using this ctDNA test in place of CEA for monitoring cases for recurrence, where a positive result at any time during surveillance triggers imaging.^37^ Applying the URL threshold for prognostication identified the subgroup with an even higher risk for recurrence (10‐ vs. 5‐fold increase). Thus, when using this test while monitoring cases, one could consider personalizing surveillance based on detection of any signal especially during the first 12 months following completion of therapy, but also triggering an earlier‐than‐scheduled CT scan whenever the result exceeded the URL.

## 5 CONCLUSIONS

This study shows that *BCAT1/IKZF1* methylation testing after completing initial treatment with curative intent predicts recurrence‐free survival, and thus identifies patients at high risk of recurrence. Identification of such patients by means of assaying for circulating DNA methylated in *BCAT1/IKZF1* does not require genomic analysis of the tumor tissue. Personalized surveillance of a more intensive and/or prolonged nature than is current clinical practice, seems warranted for patients treated for CRC with curative intent but who are positive for methylated *BCAT1/IKZF1*. Studies should also be undertaken to determine if detection of these markers after completion of all planned initial treatment warrants additional adjuvant therapy.

## AUTHOR CONTRIBUTIONS

Study design: SKP, GPY, ELS, LCL. Analysis: SKP, LCL, GPY. Writing: SKP, GPY. Data curation: GPY, AMR, KJC, ELS. Data retrieval: SKP, ELS.

## FUNDING INFORMATION

Funded in part by Clinical Genomics Pty. Ltd and Cancer Australia through The Priority‐driven Collaborative Cancer Research Scheme (Grant Number 1161720). The contents of the published material are solely the responsibility of the authors and their institutions and do not reflect the views of Cancer Australia.

## CONFLICT OF INTEREST

GPY is consultant for Clinical Genomics. SKP, LCL are paid employees of Clinical Genomics. SKP, LCL and GPY hold shares in Clinical Genomics directly or in trust. No other conflict of interest was declared by the other authors.

## Data Availability

The data that support the findings of this study are available from the corresponding author upon reasonable request.
